# Human meniscus cells express hypoxia inducible factor-1α and increased SOX9 in response to low oxygen tension in cell aggregate culture

**DOI:** 10.1186/ar2267

**Published:** 2007-07-18

**Authors:** Adetola B Adesida, Lisa M Grady, Wasim S Khan, S Jane Millward-Sadler, Donald M Salter, Timothy E Hardingham

**Affiliations:** 1 UK Centre for Tissue Engineering (UKCTE) and The Wellcome Trust Centre for Cell-Matrix Research, Faculty of Life Sciences, The University of Manchester, Michael Smith Building, Oxford Road, Manchester, M13 9PT, UK; 2 CellCoTec, Professor Bronkhorstlaan 10-D, Bilthoven 3723 MB, The Netherlands; 3 The University of Edinburgh, Queens Medical Research Inst, Little France Crescent, Edinburgh, EH16 4TJ, UK; 4 Division of Regenerative Medicine, University of Manchester, Oxford Road, Manchester M13 9PT, UK

## Abstract

In previous work we demonstrated that the matrix-forming phenotype of cultured human cells from whole meniscus was enhanced by hypoxia (5% oxygen). Because the meniscus contains an inner region that is devoid of vasculature and an outer vascular region, here we investigate, by gene expression analysis, the separate responses of cells isolated from the inner and outer meniscus to lowered oxygen, and compared it with the response of articular chondrocytes. In aggregate culture of outer meniscus cells, hypoxia (5% oxygen) increased the expression of type II collagen and SOX9 (Sry-related HMG box-9), and decreased the expression of type I collagen. In contrast, with inner meniscus cells, there was no increase in SOX9, but type II collagen and type I collagen increased. The articular chondrocytes exhibited little response to 5% oxygen in aggregate culture, with no significant differences in the expression of these matrix genes and SOX9. In both aggregate cultures of outer and inner meniscus cells, but not in chondrocytes, there was increased expression of collagen prolyl 4-hydroxylase (P4H)α(I) in response to 5% oxygen, and this hypoxia-induced expression of P4Hα(I) was blocked in monolayer cultures of meniscus cells by the hypoxia-inducible factor (HIF)-1α inhibitor (YC-1). In fresh tissue from the outer and inner meniscus, the levels of expression of the HIF-1α gene and downstream target genes (namely, those encoding P4Hα(I) and HIF prolyl 4-hydroxylase) were significantly higher in the inner meniscus than in the outer meniscus. Thus, this study revealed that inner meniscus cells were less responsive to 5% oxygen tension than were outer meniscus cells, and they were both more sensitive than articular chondrocytes from a similar joint. These results suggest that the vasculature and greater oxygen tension in the outer meniscus may help to suppress cartilage-like matrix formation.

## Introduction

The meniscus serves as a critical fibrocartilaginous tissue in the biomechanics of the knee joint, and it plays an important role in load distribution and joint stability [1,2]. Its biomechanical importance is further highlighted by the high incidence of osteoarthritis after menisectomy [3-8]. The function of the meniscus is reflected in its cellular and biochemical composition, which ensures that shear, tensile and compressive forces are appropriately distributed in the knee joint [9]. The meniscus exhibits regional and zonal variations in its cellular composition [9-13], reparative capacity [14,15] and microstructure [16,17]. The cells of the outer one-third are fibroblast-like, with extensive cellular processes that may stain positively for CD34 and are within a dense connective tissue, which is composed predominantly of type I collagen fibre bundles aligned in the circumferential direction of the tissue, along with smaller amounts of proteoglycans and minor collagens including types III and V [16,18-21]. In contrast, cells from the middle and inner portions, accounting for the remaining two-thirds of the tissue, are with few processes [17,22] and are negative for CD34 [21]. These cells have been termed fibrochondrocytes [17] and are surrounded by an extracellular matrix that is composed of collagen types I and II [17-19], with a higher content of aggrecan than in the outer region [22-24]. Based on morphological differences, the cells of the tissue have been further divided into three to four distinct populations [12].

The presence of type II collagen and aggrecan in the inner meniscus shows that this region has some similarities with articular cartilage [18-20,25]. However, the type II collagen in the meniscus is organized in a close network with collagen I fibres, which is in contrast to its diffuse fine fibre distribution in articular cartilage [19]. Further regional differences within the meniscus include the presence of vascular and neural components in the outer meniscus, which are absent from the inner region [15,26]. Perhaps as a consequence of the lack of blood supply, the reparative and regeneration potential of the inner meniscus is more limited than that of the outer region [14,27].

Cell-based tissue engineering strategies have been proposed to aid repair and to generate a meniscus substitute for implantation [13,28-32]. Meniscus cells may be appropriate for this strategy. However, during monolayer expansion of human meniscus cells there is increased expression of type I collagen and decreased expression of type II collagen, similar to the de-differentiation in culture of chondrocytes [13].

Several investigators have exploited low oxygen tension during *in vitro *culture of chondrocytes as a strategy to restore differentiated phenotype [33-37]. This stems from the fact that conventional cell culture is performed in an atmosphere containing 20% oxygen tension, whereas cartilage *in vivo*, being avascular, has much lower oxygen tension (1% to 7%) [38-41]. We recently showed that the matrix-forming phenotype of cultured primary human meniscus cells was enhanced in lowered oxygen (5%) [42,43], but the responses of cells isolated from the outer and inner regions were not investigated separately.

Recent studies have distinguished cells and tissue from the outer and inner regions of the meniscus by showing that cartilaginous marker genes, namely type II collagen and aggrecan, both exhibited significantly higher expression in cells or tissues derived from the inner region relative to cells or tissues from the outer meniscus [23,24].

The objective of the current investigation was to determine whether hypoxia inducible factor (HIF)-1α and downstream target genes that are involved in the adaptive response of cells and tissues to low oxygen tension were expressed differently in cells in the outer and inner regions of the human meniscus [44-48]. We also wished to determine whether the cells isolated from the outer and inner meniscus in culture differed in their response to lowered oxygen tension.

## Materials and methods

### Human meniscus and cartilage tissue source and cell isolation

Human articular cartilage and meniscus was obtained, with informed consent and local ethical approval (Ethics Committee of South Manchester Health Care Trust), during total knee arthroplasty from seven patients (mean age 59 years, range 36 to 77 years) with osteoarthritis. The meniscus tissue was from intact samples of medial and lateral meniscus.

The tissue was cut into small pieces within 6 hours of surgery, before overnight digestion at 37°C with 0.2% (weight/vol) collagenase II (Worthington Biochemical Corp., Reading, UK) in Dulbecco's modified Eagles medium (DMEM) containing 10% foetal calf serum (FCS). In addition, fresh tissue pieces from the inner and outer regions of samples of intact lateral meniscus were digested with collagenase, as described above, or preserved in RNAlater (Qiagen Ltd, Crawley, UK) for gene expression analysis. Tissue from the inner and outer regions represented pieces taken from about two-third and one-third of the radial distance, respectively. Isolated meniscus cells were seeded in a 75 cm^2 ^tissue culture flask at 1 × 10^4 ^cells/cm^2 ^in a humidified atmosphere under 20% oxygen and 5% carbon dioxide at 37°C in DMEM. Cells were cultured in DMEM supplemented with 10% FCS, 100 units/ml penicillin and 100 units/ml streptomycin, with added L-glutamine (2 mmol/l; all from Cambrex, Wokingham, UK). The media was changed every 2 days, and on reaching confluence (within 2 weeks) the cells were passaged (passage one) into a 225 cm^2 ^tissue culture flask. The cells were used in experiments at passage two or three of monolayer culture. Human chondrocytes were isolated from articular cartilage (obtained from the same individuals who donated menisci) by a sequential trypsin/collagenase digestion and also used in experiments at passage two or three of monolayer culture in DMEM with 10% FCS, 100 units/ml penicillin and 100 units/ml streptomycin (all from Cambrex, Wokingham, UK).

### Three-dimensional cell aggregate culture

Aggregates of second or third passage outer and inner meniscus cells or articular chondrocytes (5 × 10^5 ^cells per aggregate) were formed by centrifugation at 1,200 rpm for 5 min in a 15 ml conical culture tube. The cell aggregates were cultured for 14 days in a humidified atmosphere under conditions of normoxia (95% air and 5% carbon dioxide [20% oxygen]) or hypoxia (5% oxygen, 5% carbon dioxide and 90% nitrogen) at 37°C in DMEM containing 10% FCS and chondrogenic medium. The chondrogenic medium was composed of the following [49]: ITS+1, dexamethasone (10 nmol/l) and ascorbate-2-phosphate (25 μg/ml; all from Sigma, Poole, UK), and transforming growth factor-β_3 _(10 ng/ml; R&D Systems, Abingdon, UK).

### Meniscus cell incubation with hypoxia inducible factor-1α inhibitor (YC-1)

Cells cultured from whole meniscus at passage two were seeded onto a 12-well plate in DMEM with 10% FCS at 1 × 10^4 ^cells per well. The cells were allowed to adhere overnight under normoxia. HIF-1α inhibitor, namely 3-(5'-hydroxymethyl-2'furyl)-1-benzyl indazole (YC-1; Calbiochem, Nottingham, UK), in dimethylsulphoxide was added to DMEM with FCS at a final concentration of 1 to 50 μmol/l and incubated with meniscus cells for 5 days under normoxic and hypoxic conditions. Control monolayer cultures were incubated with DMEM containing FCS and vehicle alone (dimethylsulphoxide; 0.6% vol/vol). The growth medium was changed every 2 days.

### Gene expression analysis

Total RNA was prepared from meniscus tissue, monolayer cells and cell aggregate cultures using Tri-Reagent (Sigma, Poole, UK). To minimize changes in gene expression, cultures caps were closed before removal from the low oxygen tension incubator, and cell aggregates were immediately (<1 min) transferred into Tri-Reagent. Total RNA from fresh tissue was isolated after homogenization with a Braun mikrodismembranator (Biotech, Melsungen, Germany). Cell aggregate cultures were ground up in Tri-Reagent using Molecular Grinding Resin (Geno Technology Inc, St Louis, MO, USA). For gene expression analysis, cDNA was derived from 10 to 100 ng total RNA using global amplification [50]. Samples were diluted 1:1000 and a 1 μl aliquot was amplified by polymerase chain reaction in a 25 μl reaction volume in an MJ Research Opticon 2 real-time thermocycler using a SYBR Green Core Kit (Eurogentec, Seraing, Belgium) with gene-specific primers designed using ABI Primer Express software (Applied Biosystems, Foster City, CA, USA). Relative expression levels were normalized to β-actin mRNA expression and calculated using the 2^-ΔCt ^method [51]. All primer concentrations were 300 nmol/l unless stated otherwise. All primers were from Invitrogen (Paisley, UK) and were designed based on human sequences as summarized in Table 1.

**Table 1:** Primers used in the present study

Primer	Sequence
β-actin	Forward 5'-3' AAGCCACCCCACTTCT-CTCTAA
	Reverse 5'-3' AATGCTATCACCTCCCCTGTGT
COL1A2	Forward 5'-3'TTGCCCAAAGTT-GTCCTCTTCT
	Reverse 5'-3' AGCTTCTGTGGAACCATGGAA
COL2A1	Forward 5'-3' CTGCAAAATAAAATCTCGGTGTTCT
	Reverse 5'-3' GGGCATTTGACTCACACCAGT
HIF-1α	Forward 5-3' GTAGTTGTGGAAGT-TTATGCTAATATTGTGT
	Reverse 5'-3' CTTGTTTACAGTCTGCTCA-AAATATCTT
P4Hα(I)	Forward 5'-3' GCAGGGTGGTAATATTGGCATT
	Reverse 5'-3' AAATCAATTCCCTCATCACTGAAAG,
P4Hα(II)	Forward 5'-3'TTAGCTGTCTAGCGCCTAGCAA
	Reverse 5'-3' TTTGGTTCACTGAAACA-TCTCACA
P4Hα(III)	Forward 5'-3' CTCAACAGTCTCAGGTTCGATCA
	Reverse 5'-3' TTCTTGGTCCCTGTGGTCAAG
PHD2	Forward 5'-3'TGGCC-TATATGTGTTTAATCCTGGTT
	Reverse 5'-3'TGTTTTACAGCTGGTTAATGTG-TTGA
SOX9	Forward 5'-3'CTTTGGTTTGTGTTCGTGTTTTG
	Reverse 5'-3'AGAGAAAGAAAAAGGGAAAGGTAAGTTT

COL1A2, collagen type I alpha 2; COL2A1, collagen type II alpha 1; HIF, hypoxia inducible factor; P4H, prolyl 4-hydroxylase; PHD, HIF prolyl hydroxylase; SOX, Sry-related HMG box-9.

## Results

### HIF-1α, PHD2 and P4Hα(I) expression in inner and outer meniscus

**Figure 1:**
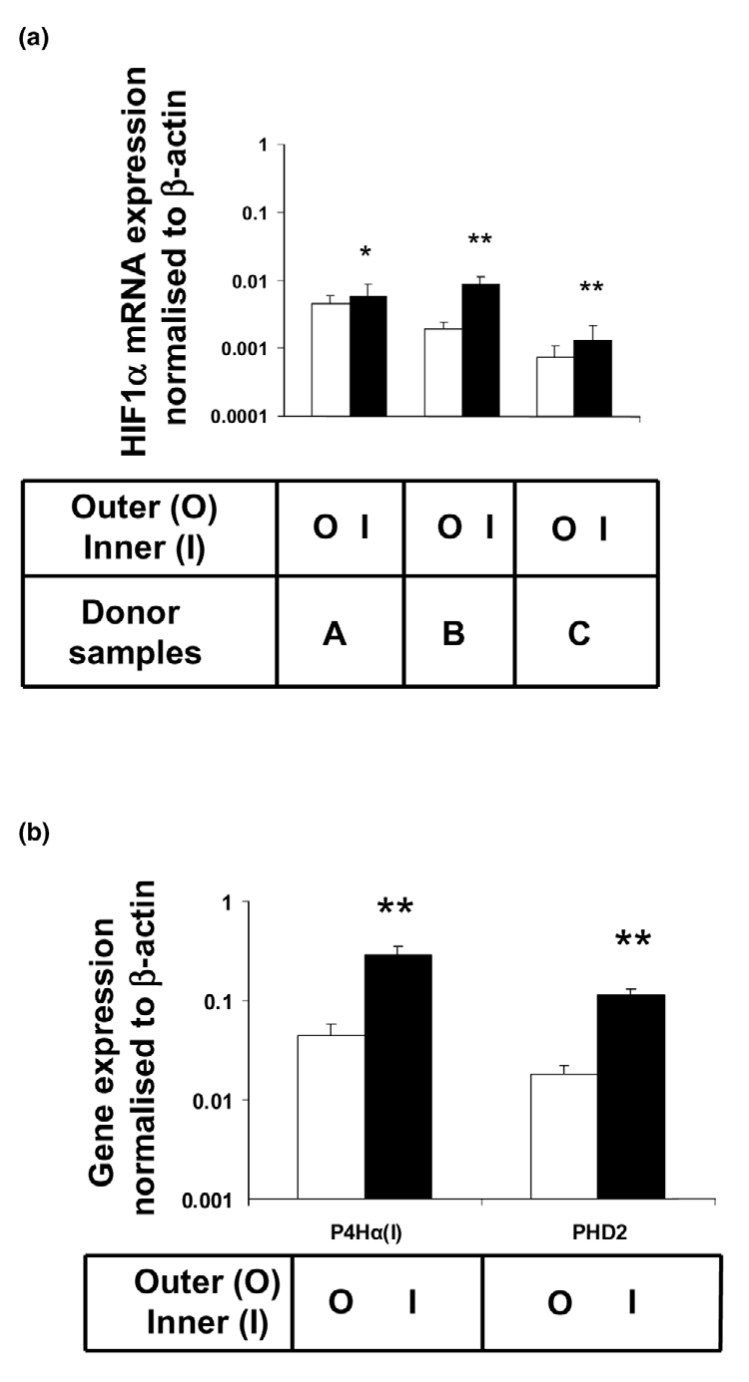
Gene expression of HIF-1α and target genes in the outer and inner meniscus. **(a) **Hypoxia inducible factor (HIF)-1α gene expression in the outer (O; white bars) and inner (I; black bars) region of meniscus tissue from three donors (*n *= 3). **(b) **Prolyl 4-hydroxylase (P4H)α(I) and HIF prolyl-hydroxylase (PHD)2 gene expression in the outer (O; white bars) and inner (I; black bars) region of meniscus tissue (*n *= 3). Values are expressed as mean ± standard deviation. *P < 0.05, **P < 0.01 (by Student's unpaired *t*-test) in inner versus outer region of the meniscus matched against the same donor.

Expression levels of a panel of genes that are involved in cellular responses to low oxygen conditions were determined. The results showed that there was significantly higher expression of HIF-1α (1.3- to 5.0-fold; *P *< 0.05 to *P *< 0.01), albeit with donor variability (Figure 1a); higher expression of HIF prolyl-hydroxylase (PHD)2 (5-fold; *P *< 0.01); and higher expression of prolyl 4-hydroxylase (P4H)α(I) (6-fold; *P *< 0.01) in samples from the inner region compared with the outer region of the meniscus (Figure 1b). The inner region cells thus exhibited evidence of gene expression induced by low oxygen tension, which was absent from the outer region.

In order to determine whether these differences in expression *in vivo *were reflected in different responses inherent in the cells present in inner and outer meniscus, we isolated the cells from the outer and inner regions and expanded them in monolayer for two to three passages. At this stage the cells were fibroblastic in morphology, and the expression of type I collagen had increased and that of type II collagen had fallen very low (data not shown). To determine the effects of lowered oxygen on their matrix-forming ability, the cells from inner and outer meniscus were cultured separately in three-dimensional cell aggregates in chondrogenic medium in the presence of 5% or 20% oxygen for 14 days, and gene expression changes in type I collagen (COL1A2 [collagen type I alpha 2]), type II collagen (COL2A1 [collagen type II alpha 1]) and SOX9 (Sry-related HMG box-9) were determined. Similar parallel experiments were performed with articular chondrocytes, so that the response of the three cell types to low oxygen tension culture could be compared.

**Figure 2:**
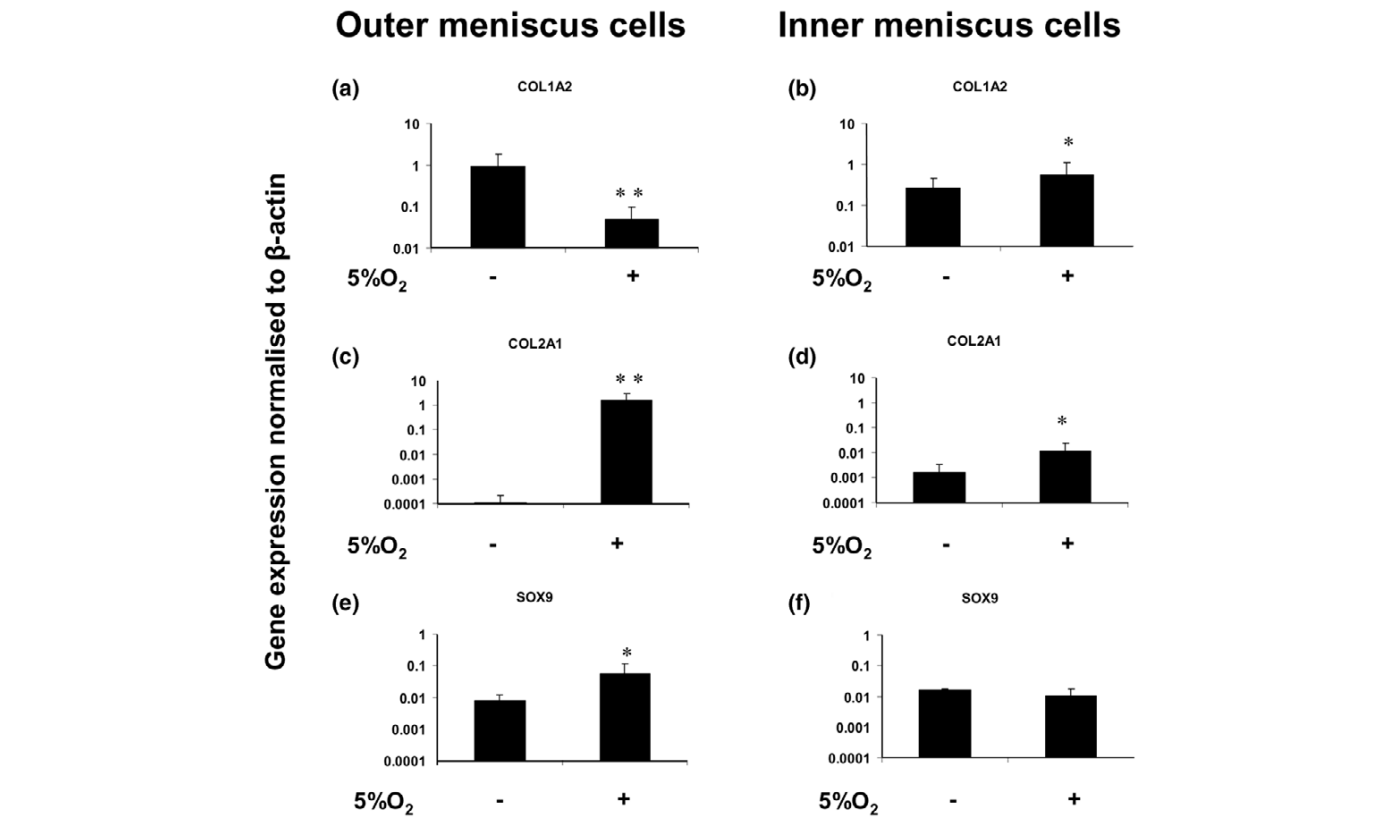
Collagen types I and II, and SOX9 gene expression in human meniscus cells. **(a, b) **Collagen type I alpha 2 (COL1A2) gene expression levels of cell aggregate cultures of (panel a) outer and (panel b) inner meniscus cells (*n *= 6) in 5% oxygen versus 20% oxygen. **(c, d) **Collagen type II alpha 1 (COL2A1) gene expression in aggregate cultures of (panel c) outer and (panel d) inner meniscus cells (*n *= 6) in 5% oxygen versus 20% oxygen. **(e, f) **SOX9 (Sry-related HMG box-9) gene expression levels of aggregate cultures of (panel e) outer and (panel f) inner meniscus cells (*n *= 6) in 5% oxygen versus 20% oxygen. *P < 0.05, **P < 0.01 (by Student's unpaired *t*-test).

**Figure 3:**
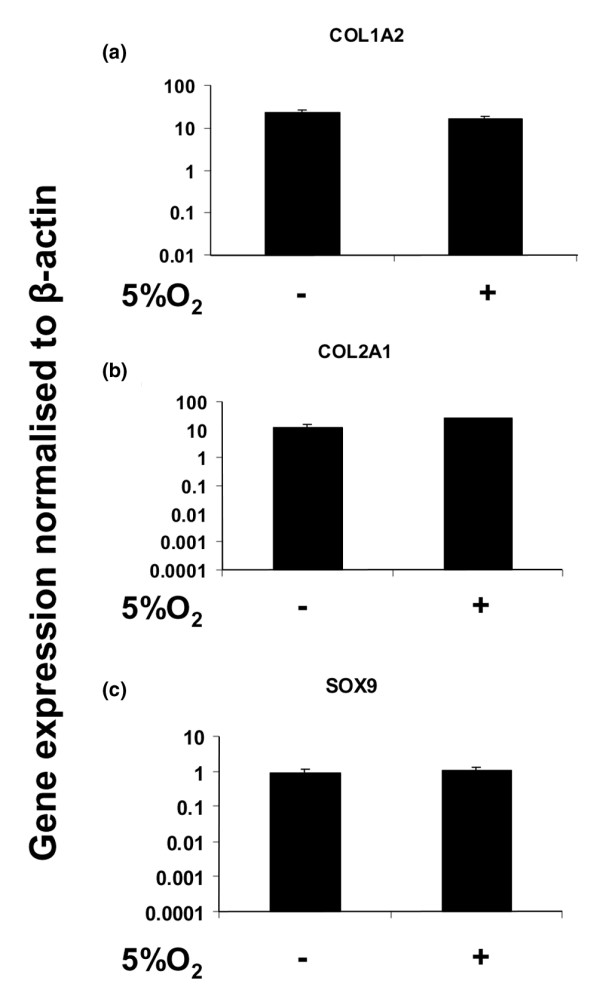
Collagen types I and II, and SOX9 gene expression in human articular chondrocytes. **(a) **Collagen type I alpha 2 (COL1A2) gene expression levels of aggregate cultures of articular chondrocytes (*n *= 3) in 5% oxygen versus 20% oxygen. **(b) **Collagen type II alpha 1 (COL2A1) gene expression in aggregate cultures of articular chondrocytes (*n *= 3) in 5% oxygen versus 20% oxygen. **(c) **SOX9 (Sry-related HMG box-9) gene expression levels of cell aggregate cultures of articular chondrocytes (*n *= 3) in 5% oxygen versus 20% oxygen. Student's unpaired *t*-test. Data are expressed as mean ± standard deviation (*n *= 3).

After 14 days in 5% oxygen, cells isolated from the outer meniscus exhibited a decrease in the expression of type I collagen by 18-fold (*P *< 0.01) as compared with 20% oxygen (Figure 2a), whereas in cells from the inner meniscus type I collagen increased 2-fold (*P *< 0.05; Figure 2b). In a parallel experiment with articular chondrocytes, the expression of type I collagen was unchanged in 5% oxygen (Figure 3a).

Cells from the outer meniscus expressed type II collagen at a very low level in monolayer culture (data not shown), and when they were transferred to aggregate culture in 5% oxygen they exhibited a very large increase (15,300-fold; *P *< 0.01) in its expression (Figure 2c). In contrast, the expression of type II collagen was higher in inner meniscus cells in monolayer than in outer meniscus cells (data not shown), but it increased only 7-fold (*P *< 0.05; Figure 2d) in aggregate cultures in 5% oxygen. The type II collagen response to lowered oxygen was thus greater in the cells cultured from the outer meniscus. The cells from the outer meniscus also exhibited a greater increase in SOX9 expression (7-fold; *P *< 0.05) in aggregate culture, whereas there was no increase in SOX9 in cells from the inner meniscus (Figure 2e,f). Under similar conditions in aggregate culture of articular chondrocytes in 5% oxygen, the expression of type II collagen and SOX9 was unchanged (Figure 3b,c).

### Induction of collagen prolyl 4-hydroxylases

**Figure 4:**
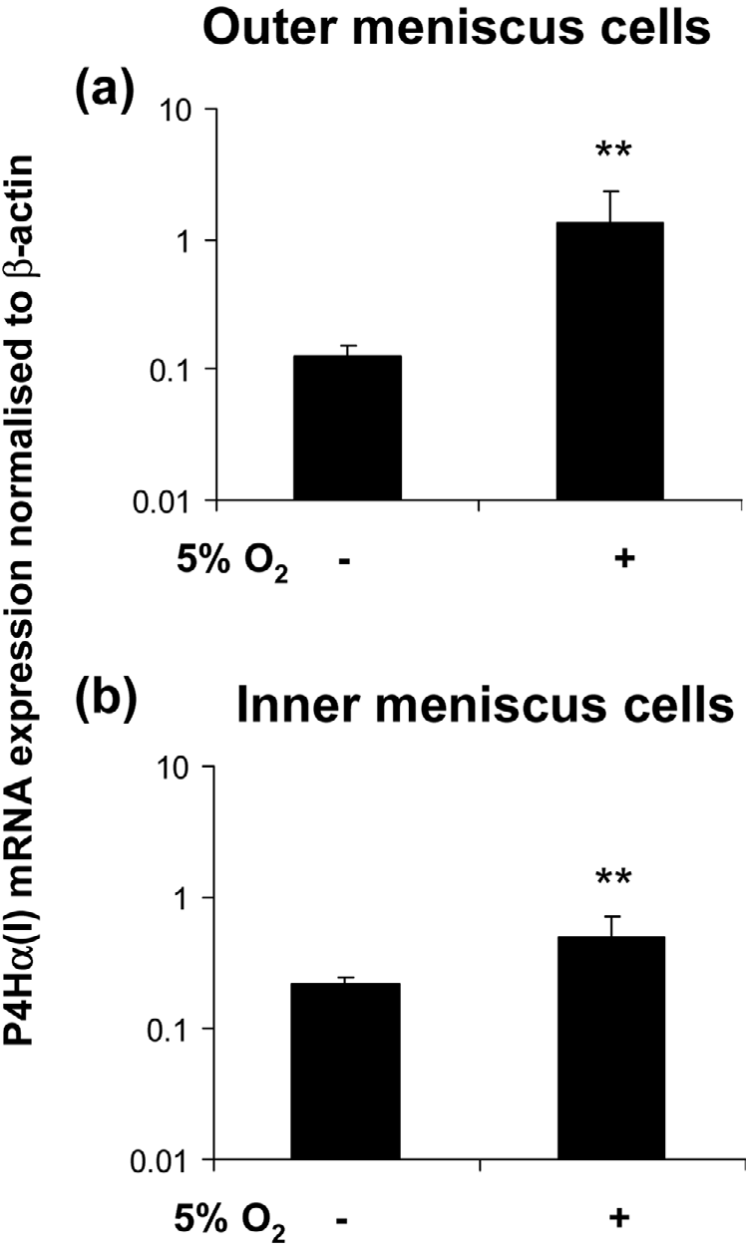
Collagen P4Hα(I) gene expression in meniscus cell aggregates. Prolyl 4-hydroxylase (P4H)α(I) gene expression in **(a) **outer meniscus cell aggregates (*n *= 4) and **(b) **inner meniscus cell aggregates (*n *= 4) in 5% oxygen versus 20% oxygen. Data are expressed as mean ± standard deviation (*n *= 3). **P < 0.01 (by Student's unpaired *t*-test).

**Figure 5:**
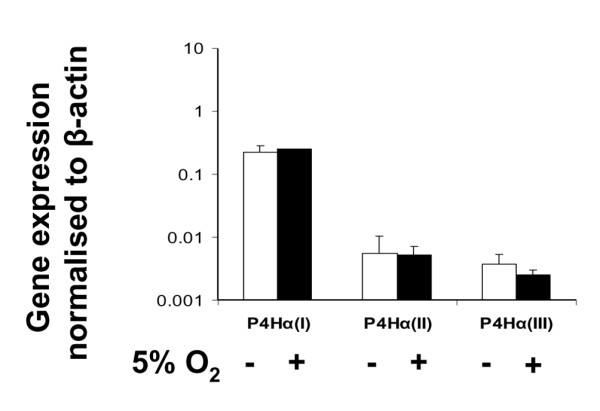
Collagen P4H isoenzyme gene expression in articular chondrocytes aggregates. Prolyl 4-hydroxylase (P4H)α isoenzyme gene expression in cell aggregates of articular chondrocytes in 20% oxygen (white bars) and 5% oxygen (black bars). Data are expressed as mean ± standard deviation (*n *= 3).

Cellular adaptation to low oxygen tension in many cells is regulated by HIF-1, a heterodimer of HIF-1α and HIF-1β, which induces the transcription of a variety of hypoxia inducible genes. We therefore investigated the expression of a known HIF-1 target gene, namely that encoding collagen P4Hα (types I, II and III), which is essential in collagen post-translational processing and fibril formation. The cells from the outer meniscus again exhibited a greater response to 5% oxygen than did the cells from the inner meniscus. The expression of P4Hα(I) isoenzyme was significantly increased (10.6-fold; *P *< 0.01) in outer meniscus cells (Figure 4a) as compared with the 2.2-fold (*P *< 0.05) increase in inner meniscus cells (Figure 4b). The expression of the other two isoenzymes of P4Hα were unaffected in 5% oxygen (data not shown), and the expression of P4Hα(I) (and that of P4Hα(II) and P4Hα(III)), under similar conditions, was unaltered in articular chondrocytes (Figure 5).

### HIF-1α inhibitor, YC-1, blocks hypoxia induced expression of P4Hα(I) in human meniscus cells

**Figure 6:**
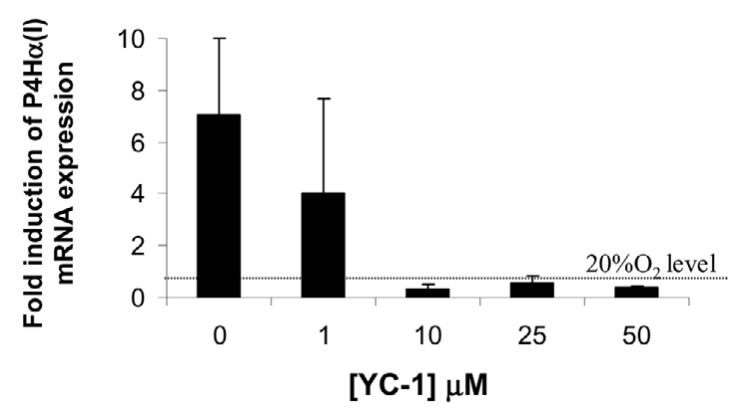
YC-1 mediated inhibition of hypoxic induction of P4H in meniscus cell aggregates. Ratio of hypoxia (5% oxygen) induced prolyl 4-hydroxylase (P4H)α(I) gene expression (post-normalization to β-actin) to P4Hα(I) gene expression (post-normalization to β-actin) in 20% oxygen in monolayer culture of meniscus cells in the presence and absence of the hypoxia inducible factor-1α inhibitor YC-1. Data are expressed as mean ± standard deviation (*n *= 3).

It has previously been established that the expression of P4Hα(I) is susceptible to transcriptional control by HIF-1α in low oxygen tension [52]. We therefore investigated the link between HIF-1α and P4Hα(I) by using the HIF-1α inhibitor YC-1 [53,54]. In monolayer cultures of a mixed population of human meniscus cells in the presence and absence of 5% oxygen tension, YC-1 inhibited the induction of P4Hα(I) in a dose-dependent manner down to the level of P4Hα(I) expression at 20% oxygen tension (Figure 6). This represents evidence that the meniscus cells upregulated HIF-1α transcriptional activity in response to 5% oxygen tension, and that this induced an increase in P4Hα(I) expression.

## Discussion

The lack of vasculature in the inner meniscus suggests that the resident cells exist in a hypoxic environment relative to meniscus cells in the outer meniscus. The results of the gene expression analysis provide new data on region-specific differences in mRNA expression in a panel of genes that are susceptible to transcriptional regulation by HIF-1α in human meniscus. Furthermore, it supports the use of gene expression to distinguish tissues and cells from different regions of the meniscus. In addition, the study provides, for the first time, data on the response of cells isolated from the inner and outer meniscus regions to low oxygen tension in culture. It was interesting that cells isolated from the outer meniscus were relatively more responsive to 5% oxygen tension than were inner meniscus cells, based on the large modulation in gene expression of collagen types I and II, SOX9 and P4Hα(I). Furthermore, it was particularly interesting that in contrast to the response of outer meniscus cell aggregates, increased SOX9 expression did not accompany the upregulated expression of type II collagen in aggregate culture of inner meniscus cells in 5% oxygen tension.

The level of type II collagen expression in aggregate culture of inner meniscus cells at 20% oxygen tension is consistent with previous reports [19,23,24], which found that the inner meniscus exhibits a more chondrocytic phenotype than does the outer meniscus. The differential induction of SOX9 seen here in response to low oxygen tension suggested that SOX9 is a necessary transcription factor of type II collagen synthesis, but that it acts in conjunction with other factors, such as SOX5 and SOX6, which are known enhancers of SOX9 [55,56]. It also suggests that increased SOX9 expression does not always correlate with type II collagen expression, and this is consistent with the findings of a previous report in articular chondrocytes [57]. Nevertheless, there were unquestionable differences between the responses of aggregate cultures of articular chondrocytes and meniscus cells (regardless of the region of cell isolation) to 5% oxygen tension. The expression of the genes studied here was clearly not modulated in aggregate cultures of articular chondrocytes by 5% oxygen tension. This may therefore reflect a greater sensitivity of meniscus cells to oxygen tension. Naturally, articular chondrocytes exist in a completely avascular microenvironment, and an oxygen tension lower than 5% may be required to elicit an hypoxic response in these cells.

This study shows that P4Hα(I) was induced by 5% oxygen tension in aggregate culture of meniscus cells, regardless of the region of origin. In contrast, aggregate cultures of articular chondrocytes exhibited no comparable induction of P4Hα. Furthermore, the upregulation of P4Hα(I) suggested that the response of meniscus cells to low oxygen tension is mediated by HIF-1α [52], and this was confirmed by its inhibition by the HIF-1α inhibitor YC-1 [53,54]. Previous studies have demonstrated YC-1 to block the expression of HIF-1α and HIF-1α regulated genes in the presence of soluble guanylyl cyclase inhibitors [54]. This strongly suggests that soluble guanylyl cyclase/cGMP signal transduction does not mediate the HIF-1α induction of P4Hα(I).

To determine whether the *in vitro *response of meniscus cells to hypoxia was relevant to their behaviour *in vivo*, we analyzed intact meniscus tissue and found higher expression of HIF-1α, P4Hα(I) and PHD2 in the inner region of the meniscus tissue as compared with the outer region. The pattern of expression correlated with the reported lower vascularity of the inner meniscus and a potentially more hypoxic microenvironment. The differential level of the constitutive expression of HIF-1α and its target genes between the meniscus regions may thus reflect a mechanism that regulates the matrix-forming phenotype of the inner meniscus. The differential expression of HIF-1α seen here is of particular interest, because the action of HIF-1α is modulated at the post-translational level. Furthermore, HIF-1α has been shown to bind to CREB (cAMP-response element-binding protein)-binding protein/p300, which SOX9 utilizes to exert its cartilage-specific type II collagen gene promoter activity [58,59]. These results suggest that the combination of the upregulation of SOX9, which activates type II collagen transcription in chondrogenic cells, and low oxygen induced upregulation of P4Hα(I) may enhance the expression of type II collagen in human meniscus cells.

## Conclusion

We demonstrate for the first time that cells isolated from the outer and inner regions of the meniscus respond differentially to lowered oxygen tension (5% oxygen). Based on the large modulation in gene expression of the panel of genes (collagen types I and II, SOX9 and P4Hα(1)) investigated in this study, it appears that cells from the outer meniscus are relatively more responsive to lowered oxygen tension than are their inner counterparts. Furthermore, the results show gene expression analysis to be a powerful tool in distinguishing tissue or cells from the outer and inner meniscus, and further extend the repertoire of genes that are constitutively and differentially expressed within specific regions of the meniscus. Most importantly, our findings revealed that HIF1α and downstream target genes PHD2 and P4Hα were upregulated in the inner meniscus relative to the outer meniscus, and that the response of meniscus cells (regardless of the region of cell isolation) to 5% oxygen tension was mediated by HIF-1α. Collectively, our data suggest that hypoxia driven expression of HIF-1α may be important in determining the phenotype of the inner meniscus.

## Abbreviations

DMEM = Dulbecco's modified Eagle's medium; FCS = foetal calf serum; HIF = hypoxia inducible factor; P4H = prolyl 4-hydroxylase; PHD = HIF prolyl-hydroxylase; SOX9 = Sry-related HMG box-9.

## Competing interests

The authors declare that they have no competing interests.

## Authors' contributions

ABA conceived, designed and executed the experiments described in this study, and was responsible for writing the initial versions of the manuscript. LMG and SJMS performed RNA isolation and cell culture experiments included in this manuscript. WSK was responsible for tissue procurement and processing. DMS and TEH supervised and oversaw the completion of the studies as well as the writing of the manuscript.

## References

[B1] Ahmed AM, Mow VC, Arnoczky SP, Jackson DW (1992). The load-bearing role of the knee meniscus. Knee Meniscus: Basic and Clinical Foundations.

[B2] Levy IM, Torzilli PA, Fisch ID, Mow VC, Arnoczky SP, Jackson DW (1992). The contribution of the menisci to the stabilty of the knee. Knee Meniscus: Basic and Clinical Foundations.

[B3] Fairbank T (1948). Knee joint changes after menisectomy. J Bone Joint Surg.

[B4] Aagaard H, Verdonk R (1999). Function of the normal meniscus and consequences of meniscal resection. Scand J Med Sci Sports.

[B5] Wyland DJ, Guilak F, Elliott DM, Setton LA, Vail TP (2002). Chondropathy after meniscal tear or partial meniscectomy in a canine model. J Orthop Res.

[B6] Roos H, Adalberth T, Dahlberg L, Lohmander LS (1995). Osteoarthritis of the knee after injury to the anterior cruciate ligament or meniscus: the influence of time and age. Osteoarthritis Cartilage.

[B7] Cox JS, Cordell LD (1977). The degenerative effects of medial meniscus tears in dogs' knees. Clin Orthop Relat Res.

[B8] Cox JS, Nye CE, Schaefer WW, Woodstein IJ (1975). The degenerative effects of partial and total resection of the medial meniscus in dogs' knees. Clin Orthop Relat Res.

[B9] Proctor CS, Schmidt MB, Whipple RR, Kelly MA, Mow VC (1989). Material properties of the normal medial bovine meniscus. J Orthop Res.

[B10] Ghadially FN, Thomas I, Yong N, Lalonde JM (1978). Ultrastructure of rabbit semilunar cartilages. J Anat.

[B11] McDevitt CA, Miller RR, Spindler KP, Mow VC, Arnoczky SP, Jackson DW (1992). The cells and cell matrix interactions of the meniscus. Knee Meniscus: Basic and Clinical Foundations.

[B12] Hellio Le Graverand MP, Ou Y, Schield-Yee T, Barclay L, Hart D, Natsume T, Rattner JB (2001). The cells of the rabbit meniscus: their arrangement, interrelationship, morphological variations and cytoarchitecture. J Anat.

[B13] Nakata K, Shino K, Hamada M, Mae T, Miyama T, Shinjo H, Horibe S, Tada K, Ochi T, Yoshikawa H (2001). Human meniscus cell: characterization of the primary culture and use for tissue engineering. Clin Orthop.

[B14] Arnoczky SP, Mow VC, Arnoczky SP, Jackson DW (1992). Gross and vascular anatomy of the meniscus and its role in meniscal healing, regeneration, and remodelling. Knee Meniscus: Basic and Clinical Foundations.

[B15] Arnoczky SP, Warren RF (1982). Microvasculature of the human meniscus. Am J Sports Med.

[B16] Adams ME, Hukins DWL, Mow VC, Arnoczky SP, Jackson DW (1992). The extracellular matrix of the meniscus. Knee Meniscus: Basic and Clinical Foundations.

[B17] McDevitt CA, Webber RJ (1990). The ultrastructure and biochemistry of meniscal cartilage. Clin Orthop Relat Res.

[B18] Cheung HS (1987). Distribution of type I, II, III and V in the pepsin solubilized collagens in bovine menisci. Connect Tissue Res.

[B19] Kambic HE, McDevitt CA (2005). Spatial organization of types I and II collagen in the canine meniscus. J Orthop Res.

[B20] Roughley PJ, White RJ (1992). The dermatan sulfate proteoglycans of the adult human meniscus. J Orthop Res.

[B21] Verdonk PC, Forsyth RG, Wang J, Almqvist KF, Verdonk R, Veys EM, Verbruggen G (2005). Characterisation of human knee meniscus cell phenotype. Osteoarthritis Cartilage.

[B22] Ghadially FN, Lalonde JM, Wedge JH (1983). Ultrastructure of normal and torn menisci of the human knee joint. J Anat.

[B23] Valiyaveettil M, Mort JS, McDevitt CA (2005). The concentration, gene expression, and spatial distribution of aggrecan in canine articular cartilage, meniscus, and anterior and posterior cruciate ligaments: a new molecular distinction between hyaline cartilage and fibrocartilage in the knee joint. Connect Tissue Res.

[B24] Upton ML, Chen J, Setton LA (2006). Region-specific constitutive gene expression in the adult porcine meniscus. J Orthop Res.

[B25] Melrose J, Smith S, Cake M, Read R, Whitelock J (2005). Comparative spatial and temporal localisation of perlecan, aggrecan and type I, II and IV collagen in the ovine meniscus: an ageing study. Histochem Cell Biol.

[B26] Wilson AS, Legg PG, McNeur JC (1969). Studies on the innervation of the medial meniscus in the human knee joint. Anat Rec.

[B27] Arnoczky SP, Warren RF (1983). The microvasculature of the meniscus and its response to injury. An experimental study in the dog. Am J Sports Med.

[B28] Arnoczky SP (1999). Building a meniscus. Biologic considerations. Clin Orthop.

[B29] Adams SB, Randolph MA, Gill TJ (2005). Tissue engineering for meniscus repair. J Knee Surg.

[B30] Buma P, Ramrattan NN, van Tienen TG, Veth RPH (2004). Tissue engineering of the meniscus. Biomaterials.

[B31] Marsano A, Millward-Sadler SJ, Salter DM, Adesida A, Hardingham T, Tognana E, Kon E, Chiari-Grisar C, Nehrer S, Jakob M (2007). Differential cartilaginous tissue formation by human synovial membrane, fat pad, meniscus cells and articular chondrocytes. Osteoarthritis and Cartilage.

[B32] Sweigart MA, Athanasiou KA (2001). Toward tissue engineering of the knee meniscus. Tissue Eng.

[B33] Domm C, Schunke M, Christesen K, Kurz B (2002). Redifferentiation of dedifferentiated bovine articular chondrocytes in alginate culture under low oxygen tension. Osteoarthritis Cartilage.

[B34] Murphy CL, Sambanis A (2001). Effect of oxygen tension on chondrocyte extracellular matrix accumulation. Connect Tissue Res.

[B35] Murphy CL, Sambanis A (2001). Effect of oxygen tension and alginate encapsulation on restoration of the differentiated phenotype of passaged chondrocytes. Tissue Eng.

[B36] Murphy CL, Polak JM (2004). Control of human articular chondrocyte differentiation by reduced oxygen tension. J Cell Physiol.

[B37] Grimshaw MJ, Mason RM (2001). Modulation of bovine articular chondrocyte gene expression in vitro by oxygen tension. Osteoarthritis Cartilage.

[B38] Etherington PJ, Winlove P, Taylor P, Paleolog E, Miotla JM (2002). VEGF release is associated with reduced oxygen tensions in experimental inflammatory arthritis. Clin Exp Rheumatol.

[B39] Brighton CT, Heppenstall RB (1971). Oxygen tension in zones of the epiphyseal plate, the metaphysis and diaphysis. An in vitro and in vivo study in rats and rabbits. J Bone Joint Surg Am.

[B40] Lund-Olesen K (1970). Oxygen tension in synovial fluids. Arthritis Rheum.

[B41] Freshney R (1987). Culture of Animal Cells: a Manual of Basic Techniques.

[B42] Adesida A, Tweats L, Millward-Sadler J, Salter D, Hardingham T (2005). Cultured human meniscus cells are chondrogenic in pellet culture: this is enhanced by hypoxia and involves upregulation of prolyl 4-hydroxylase type I. Trans Orthop Res Soc.

[B43] Adesida AB, Grady LM, Khan WS, Hardingham TE (2006). The matrix-forming phenotype of cultured human meniscus cells is enhanced after culture with fibroblast growth factor 2 and is further stimulated by hypoxia. Arthritis Res Ther.

[B44] Berra E, Benizri E, Ginouves A, Volmat V, Roux D, Pouyssegur J (2003). HIF prolyl-hydroxylase 2 is the key oxygen sensor setting low steady-state levels of HIF-1alpha in normoxia. EMBO J.

[B45] Semenza G (2001). HIF-1, O_2_, and the 3 PHDs. How animal cells signal hypoxia to the nucleus. Cell.

[B46] Semenza G (2001). Hypoxia-inducible factor 1: oxygen homeostasis and disease pathophysiology. Trends Mol Med.

[B47] Semenza GL (2001). HIF-1 and mechanisms of hypoxia sensing. Curr Opin Cell Biol.

[B48] Semenza G (2002). Signal transduction to hypoxia-inducible factor 1. Biochem Pharmacol.

[B49] Barbero A, Ploegert S, Heberer M, Martin I (2003). Plasticity of clonal populations of dedifferentiated adult human articular chondrocytes. Arthritis Rheum.

[B50] Al-Taher A, Bashein A, Nolan T, Hollingsworth M, Brady G (2000). Global cDNA amplification combined with real-time RT-PCR: accurate quantification of multiple human potassium channel genes at the single cell level. Yeast.

[B51] Livak KJ, Schmittgen TD (2001). Analysis of relative gene expression data using real-time quantitative PCR and the 2-ΔΔCT method. Methods.

[B52] Takahashi Y, Takahashi S, Shiga Y, Yoshimi T, Miura T (2000). Hypoxic induction of prolyl 4-hydroxylase alpha (I) in cultured cells. J Biol Chem.

[B53] Chun Y-S, Yeo E-J, Choi E, Teng C-M, Bae J-M, Kim M-S, Park J-W (2001). Inhibitory effect of YC-1 on the hypoxic induction of erythropoietin and vascular endothelial growth factor in Hep3B cells. Biochem Pharmacol.

[B54] Chun Y-S, Yeo E-J, Park J-W (2004). Versatile pharmacological actions of YC-1: anti-platelet to anticancer. Cancer Lett.

[B55] Lefebvre V, Li P, de Crombrugghe B (1998). A new long form of Sox5 (L-Sox5), Sox6 and Sox9 are coexpressed in chondrogenesis and cooperatively activate the type II collagen gene. EMBO J.

[B56] Ikeda T, Kamekura S, Mabuchi A, Kou I, Seki S, Takato T, Nakamura K, Kawaguchi H, Ikegawa S, Chung UI (2004). The combination of SOX5, SOX6, and SOX9 (the SOX trio) provides signals sufficient for induction of permanent cartilage. Arthritis Rheum.

[B57] Aigner T, Gebhard PM, Schmid E, Bau B, Harley V, Poschl E (2003). SOX9 expression does not correlate with type II collagen expression in adult articular chondrocytes. Matrix Biol.

[B58] Ebert BL, Bunn HF (1998). Regulation of transcription by hypoxia requires a multiprotein complex that includes hypoxia-inducible factor 1, an adjacent transcription factor, and p300/CREB binding protein. Mol Cell Biol.

[B59] Tsuda M, Takahashi S, Takahashi Y, Asahara H (2003). Transcriptional co-activators CREB-binding protein and p300 regulate chondrocyte-specific gene expression via association with Sox9. J Biol Chem.

